# Encapsulation of CsPb_2_Br_5_ in TiO_2_ Microcrystals to Enhance Environmental Stability

**DOI:** 10.3390/mi14122186

**Published:** 2023-11-30

**Authors:** Yuezhu Wang, Xiaotong Xu, Wenchao Yang, Yawen Wei, Junsheng Wang

**Affiliations:** 1 Liaoning Key Laboratory of Marine Sensing and Intelligent Detection, Dalian Maritime University, Dalian 116026, China; wangyuezhu@dlmu.edu.cn (Y.W.); wangjsh@dlmu.edu.cn (J.W.); 2 College of Environmental Sciences and Engineering, Dalian Maritime University, Dalian 116026, China; 3 Key Laboratory of Coastal Ecology and Environment of State Oceanic Administration, National Marine Environmental Monitoring Center, Dalian 116023, China; wcyang@nmemc.org.cn (W.Y.); ywwei@nmemc.org.cn (Y.W.); 4 Key Laboratory of Industrial Ecology and Environmental Engineering, Dalian University of Technology, Dalian 116024, China

**Keywords:** TiO_2_, CsPb_2_Br_5_, p-aminobenzoic acid, environmental stability

## Abstract

All-inorganic lead halide perovskite has emerged as an attractive semiconducting material due to its unique optoelectronic properties. However, its poor environmental stability restricts its broad application. Here, a simple method for the fabrication of CsPb_2_Br_5_/TiO_2_ is investigated. The introduction of p-aminobenzoic acid, which has two functional groups, is critical for the capping of amorphous TiO_2_ on CsPb_2_Br_5_. After calcination at 300 °C, amorphous TiO_2_ crystallizes into the anatase phase. The CsPb_2_Br_5_/TiO_2_ NCs show high long-term stability in water and enhanced stability against ultraviolet radiation and heat treatment, owing to the chemical stability of TiO_2_. More importantly, photo-electrochemical characterizations indicate that the formation of TiO_2_ shells can increase the charge separation efficiency. Hence, CsPb_2_Br_5_/TiO_2_ exhibits improved photoelectric activity owing to the electrical conductivity of the TiO_2_ in water. This study provides a new route for the fabrication of optoelectronic devices and photocatalysts based on perovskite NCs in the aqueous phase. Furthermore, the present results demonstrate that CsPb_2_Br_5_/TiO_2_ NCs has considerable potential to be used as a photoelectric material in optical sensing and monitoring.

## 1. Introduction

Colloidal semiconductor nanocrystals (NCs) possess a unique blend of quantum size effects that effectively amplify their optical properties in comparison to their volumetric counterparts; they have emerged as a promising photoelectric material that is currently under extensive investigation. These NCs typically exhibit a size ranging from 2 to 20 nm, possess a distinctive surface chemistry, and exist in a state of colloidal freedom, enabling their dispersion in diverse solvents and substrates and ultimately facilitating their integration into a wide array of devices. Over the past few years, significant advances have been made in the field of all-inorganic perovskite nanocrystal synthesis, with extensive research being conducted in this domain. As a result, all-inorganic cesium lead halide perovskite NCs, offering narrow emission bands, excellent charge transport properties, high quantum efficiencies, and broad color tunability, have been developed [1,2,3,4]. Therefore, lead halide perovskite NCs have been regarded as the most promising materials for application in photodetectors [5], lasers [6], photovoltaics [7], and LEDs [8]. 

Although strong progress has been made in the preparation of all-inorganic perovskite nanocrystals, many challenges remain. For example, perovskites show great promise in terms of photoluminescent quantum yields and color tunability; however, the poor chemical stability of the perovskite, including poor water, photo, and thermal stability, severely limits their application. Thus, it is critical to achieve environmental stability for perovskite NCs in practical applications and highly desirable to develop methods that can prepare perovskite NCs with tunable morphologies and enhanced stabilities. In order to improve the stability, perovskite NCs have been embedded in silicone resin [9], polymer [10], or inorganic matrix [11,12,13,14], or coated with strongly binding ligands [15]. Although the chemical stability of perovskite NCs can be enhanced significantly via these methods, the insulating shells (including organics or amorphous inorganic shells) restrict charge transport for further optoelectronic or catalytic applications. 

TiO_2_ is classified as an N-type semiconductor photocatalyst, which has the advantages of high optical stability, low cost, and non-toxicity; hence, it has been widely studied in the field of energy and environment. TiO_2_ has a wide band gap energy of 3.2 eV, so photons with the appropriate energy (<400 nm) can excite electrons to jump from the valence band to the conducting band, resulting in electrons (e^−^) and holes (h^+^). Therefore, as an excellent electron collecting and transporting material, TiO_2_ is an excellent shell material to enhance the stability of perovskites. Zheng’s group has synthesized CsPbBr_3_ NCs coated with anatase TiO_2_ via hydrolysis and calcination processes, and the CsPbBr_3_/TiO_2_ NCs exhibited improved chemical stability [16]. Meanwhile, a photoelectrochemical study indicated that the formation of CsPbBr_3_/TiO_2_ could facilitate the charge transfer of CsPbBr_3_ NCs. During the formation of CsPbBr_3_/TiO_2_, tetrabutyl titanate (TBT) was added into the CsPbBr_3_ solution directly. However, perovskite NCs are easily degraded in polar environments. Thus, the strong polarity of TBT can affect the stability of perovskite NCs during the coating progress, due to their high sensitivity to the surrounding environment. In addition, this method cannot eliminate the presence of water in the fabrication of TiO_2_-coated CsPbBr_3_ NCs. In the field of photocatalysis, Tüysüz et al. fabricated a CsPbBr_3_/P25 composite for photocatalytic benzyl alcohol oxidation and proved its higher visible catalytic activity than P25 [17]. However, the existence of CsPbBr_3_ on the surface of P25 is not conducive to the stability of CsPbBr_3_. Thus, it is essential to develop a novel strategy to enhance the chemical stability of perovskite NCs as well as maintain good charge transport properties. 

Due to its unique photoelectric performance, CsPb_2_Br_5_ has attracted the attention of researchers. Compared with CsPbBr_3_ (with a 3D structure), CsPb_2_Br_5_ (with a 2D structure) shows excellent optical properties due to its sufficient gain toward whispering gallery mode lasing [18,19]. Furthermore, CsPb_2_Br_5_ displays a stronger stability against water compared with CsPbBr_3_. Nonetheless, its stability against water also limits its broad application. Although different methods have been reported to enhance the stability of perovskite NCs, all of them have focused on improving the stability of CsPbBr_3_. There is no report on how to improve the environmental stability of CsPb_2_Br_5_. 

In this study, we report a facile method to fabricate TiO_2_-coated CsPb_2_Br_5_ NCs. The ligands, palmitic acid (PA), and PABA (which have two functional groups including the carboxyl group and amino group) were introduced to the fabrication process. Titanium carboxylate was first formed by the reaction of the carboxyl group in PABA and tetrabutyl titanate (TBT). The introduction of PABA avoids the negative effect of TBT, which has strong polarity. Furthermore, the CsPb_2_Br_5_ nanocrystals can be capped by amorphous TiO_2_ (A-TiO_2_) via the interaction between the amino group and CsPb_2_Br_5_ NCs. After calcination, the A-TiO_2_ shell is crystallized into the anatase crystal phase. The formation of the protective TiO_2_ shells can isolate NCs from water, even when immersing the CsPb_2_Br_5_/TiO_2_ nanocrystals in water. Meanwhile, the photostability and thermal stability are also significantly improved. Moreover, the charge transfer and photoelectric activity in real water were also tested.

## 2. Experimental Section

### 2.1. Chemicals

Octadecene (ODE), Cs_2_CO_3_, PbBr_2_, oleylamine (OAm), oleic acid (OA), and PABA were obtained from Aladdin (Shanghai, China). PA was purchased from Tianjin Bodi Chemical Co., Ltd. (Tianjin, China) TBT and ethanol were purchased from Sinopharm Chemical Reagent Co., Ltd. (Shanghai, China). The chemicals were of analytical grade and without further purification. 

### 2.2. Preparation of CsPb_2_Br_5_/TiO_2_ NCs

To fabricate CsPb_2_Br_5_/TiO_2_ NCs, the synthesis process involved several steps. Firstly, the Cs-precursor was fabricated. As a typical procedure, Cs_2_CO_3_ (0.08 g) was loaded into a 50 mL 3-neck flask along with octadecene (3 mL) and stearic acid (0.55 g), heated for 0.5 h at 120 °C, and then heated under N_2_ to 150 °C until all Cs_2_CO_3_ was completely dissolved. Then, the product was kept in sample bottles. In order to prevent Cs-stearate from precipitating out of the ODE at room temperature, the solution needed to be preheated to 120 °C before injection. In a separate 3-necked flask, 5 mL ODE and 0.068 g PbBr_2_ were mixed together. The flask was then heated to 120 °C for 1 h under a nitrogen flow. Following this, 0.5 g palmitic acid and 0.5 mL OAm were injected into the solution. The temperature was then increased to 160 °C. At this point, 0.4 mL of the previously prepared Cs-precursor solution was quickly injected into the PbBr_2_ solution. To fabricate CsPb_2_Br_5_/TiO_2_ NCs, the TiO_2_ precursor (which was fabricated by the mixing of ODE (4 mL), TBT (0.5 mL), and PABA (0.05 g)) was injected into the solution as soon as possible after the injection of Cs-precursor. The reaction was maintained for 10 min followed by cooling in an ice bath. Then, the as-prepared crude solution was centrifuged and purified 3 times by n-hexane. The precipitate after centrifugation was dried in a vacuum at 30 °C. Finally, the product was calcined under nitrogen flow at 300 °C for 4 h to obtain the desired CsPb_2_Br_5_/TiO_2_ NCs.

### 2.3. Material Characterization

The morphologies of the samples were determined by using a Tecnai F20 S-TWIN microscope transmission electron microscope operating at 200 kV (TEM, FEI Company, Hillsboro, OR, USA). The crystal structures of the powders were investigated on a Rigaku D/MAX-2400 diffractometer (Rigaku, Tokyo, Japan) with Cu-Ka radiation, operated at 40 kV and 40 mA (scanning step: 0.02°/s) in the 2θ range of 10–80°. FT-IR spectra of the samples were characterized using the FT-IR method (NICOLET 6700, Thermo SCIENTIFIC, Waltham, MA, USA). The XPS patterns were acquired by an X-ray photoelectron spectrometer (ESCALAB™ 250Xi, Thermo Fisher, Waltham, MA, USA). The PL spectra of the perovskite NCs were obtained using the F-7000 Fluorescence Spectrophotometer (Hitachi, Hitachi City, Japan).

### 2.4. Environmental Stability Characterization

Water stability: The perovskite NCs were immersed in DI and dispersed evenly by ultrasound. The CsPb_2_Br_5_/TiO_2_ NCs with different soaking times were dried and the PL spectra were measured. 

Photostability: The perovskite NCs were subjected to ultraviolet light irradiation. The experiment involved taking powder samples at regular intervals, starting from the 12th hour and continuing until the 24th hour. These samples were later used for PL spectra testing.

Thermal stability: To investigate the influence of temperature on the luminescence properties of perovskite NCs, a series of experiments were conducted. The perovskite NCs were subjected to heating in a controlled environment with temperatures ranging from 30 to 200 °C. To obtain a comprehensive understanding of the temperature-dependent luminescence behavior, the PL spectra of the perovskite NCs were measured at regular intervals of 10 °C. This systematic approach allowed for analysis of how changes in temperature affect the emission characteristics of the perovskite NCs. By examining the PL spectra obtained at different temperatures, valuable insights into the thermal stability and performance of perovskite NCs, in terms of luminescence, can be obtained. 

## 3. Results and Discussion

**Scheme 1:**
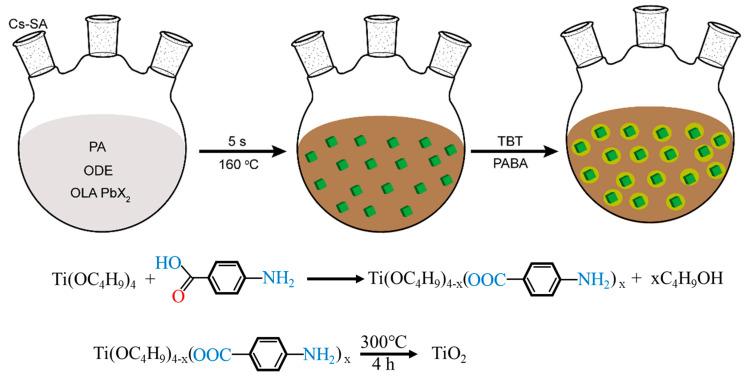
Schematic diagram illustrating the preparation of CsPb_2_Br_5_/A-TiO_2_.

**Figure 1:**
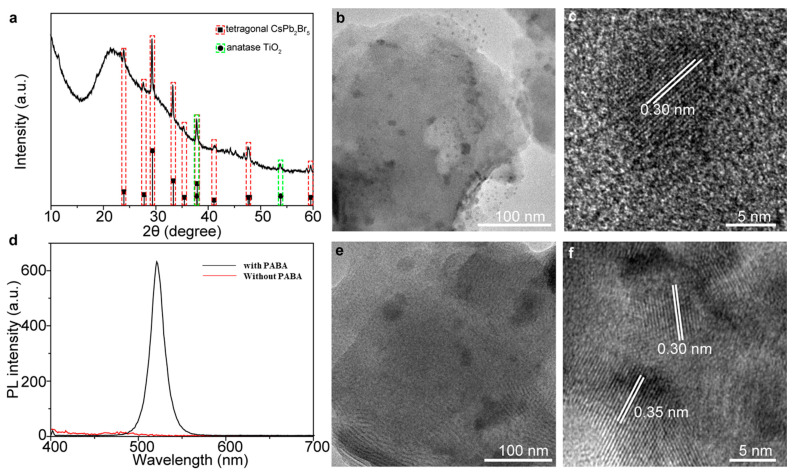
(**a**) XRD pattern of CsPb_2_Br_5_/TiO_2_, (**b**,**c**) TEM and HR-TEM images of CsPb_2_Br_5_/A-TiO_2_, (**d**) PL spectra of CsPb_2_Br_5_/TiO_2_ with and without PABA, and (**e**,**f**) TEM and HR-TEM images of CsPb_2_Br_5_/TiO_2_.

First, the TiO_2_ precursor was fabricated from ODE, TBT, and PABA. In terms of hard/soft acid/base theory [20], the -COOH tends to react with Ti^4+^ to form carboxylate. On the other hand, the -NH_2_ group in PABA does not participate in the reaction with Ti^4+^. Therefore, in the presence of carboxylate, the Ti^4+^ ions can interact with the perovskite through the -NH_2_ groups when the carboxylate is introduced into the solution of lead halide perovskite precursor at a temperature of 160 °C. This interaction between Ti^4+^ and the perovskite leads to the formation of a composite material known as A-TiO_2_. Meanwhile, the excess of PbBr_2_ is generated by the addition of carboxylate, which may impede the diffusion of PbBr_2_ in solution. Hence, CsPb_2_Br_5_ is formed by the presence of an excess of PbBr_2_ [21]. As a result, CsPb_2_Br_5_/A-TiO_2_ is synthesized (Scheme 1). After calcination for 4 h at 300 °C under a nitrogen atmosphere, A-TiO_2_ is transformed into anatase phase. EDAX was performed for the elemental analysis. The results are shown as Figure S1. The signals of Br, Pb, Cs, Ti, and O elements are clearly illustrated, indicating the formation of CsPb_2_Br_5_/TiO_2_. The XRD pattern was obtained to investigate the crystal structures of perovskite and TiO_2_. As shown in Figure 1a, diffraction peaks are indicated at 2θ 24.03 (202), 27.77 (114), 29.35 (213), 33.34 (310), 35.44 (312), 37.89 (313), 41.11 (314), 47.62 (413), 47.86 (420), and 59.55 (434), respectively. In fact, these peaks are matched well with the typical peaks belonging to the tetragonal phase of CsPb_2_Br_5_ according to JCPDS 25-0211. Further, we also found that the peaks shown at 37.80 and 53.89 are matched with (004) and (105) planes and belong to anatase TiO_2_, according to JCPDS 21-1272. It should be noted that the typical peak ascribed to the (004) plane of anatase TiO_2_ overlaps with the peak belonging to the (313) plane of the tetragonal phase of CsPb_2_Br_5_ [22]. 

The low intensity of the anatase TiO_2_ XRD peaks indicates incomplete crystallization of TiO_2_ with possible amorphous components [16]. The broad XRD peak is caused by the formation of carbon during the heating process under a nitrogen atmosphere. 

The crystal structures of pure TiO_2_ and CsPb_2_Br_5_ have been extensively studied and are presented in Figure S2. These crystal structures provide valuable insights into the properties and behaviors of these materials. The results of the study confirm that TiO_2_ adopts the anatase crystal structure, while CsPb_2_Br_5_ exhibits a tetragonal phase structure. The tetragonal phase structure of CsPb_2_Br_5_ is known for its stability and ability to withstand external pressures. 

TEM and HR-TEM were utilized in this study to investigate the evolution of CsPb_2_Br_5_ and TiO_2_. Figure 1b shows that the CsPb_2_Br_5_ NCs have a core size of around 10 nm and are embedded in a matrix. Furthermore, the HR-TEM image (Figure 1c) of the CsPb_2_Br_5_ NCs reveals that the interplanar distance is about 0.30 nm, corresponding to the (213) plane of the crystal. Meanwhile, the shell without lattice fringe can be ascribed to the formation of A-TiO_2_. In addition, the obtained sample (CsPb_2_Br_5_/A-TiO_2_) still shows strong photoluminescence under ultraviolet radiation. However, if the mixture of TBT and ODE without PABA participates in the reaction, the photoluminescence of the resulting product disappears (Figure 1d). Hence, the introduction of PABA is beneficial to reduce the negative effect of TBT polarity on perovskite NCs. After calcination at 300 °C, the interplanar distance of CsPb_2_Br_5_ is still 0.30 nm, indicating that the crystal phase of perovskite does not change (Figure 1f). Thus, we can deduce that the formation of anatase TiO_2_ has no effect on the crystal phase of the CsPb_2_Br_5_. In addition, the lattice fringe of TiO_2_ (0.35 nm) belonging to the (101) plane of anatase TiO_2_ is observed in the TEM image (Figure 1e,f). 

Selective electron diffraction (SAD) was also performed to investigate the structures of CsPb_2_Br_5_ and CsPb_2_Br_5_/TiO_2_ NCs. As shown in Figure S3, the SAD image of CsPb_2_Br_5_ revealed clear rings corresponding to the (213) plane. This indicates that the crystal structure of CsPb_2_Br_5_ was well-defined. However, the typical plane ascribed to anatase TiO_2_ was not observed in the SAD image of CsPb_2_Br_5_/TiO_2_ NCs. This result may be caused by the incomplete crystallization of TiO_2_. This observation is consistent with the XRD pattern, which also shows a lack of distinct peaks corresponding to anatase TiO_2_. The incomplete crystallization of TiO_2_ in CsPb_2_Br_5_/TiO_2_ NCs may be attributed to various factors such as the synthesis conditions or the presence of CsPb_2_Br_5_, which could inhibit the formation of TiO_2_ crystals. Further investigations are needed to fully understand the reasons behind the incomplete crystallization of TiO_2_ in CsPb_2_Br_5_/TiO_2_ NCs. By improving the crystallization of TiO_2_, the overall performance and properties of CsPb_2_Br_5_/TiO_2_ NCs can potentially be enhanced for various applications in optoelectronics and photocatalysis.

**Figure 2:**
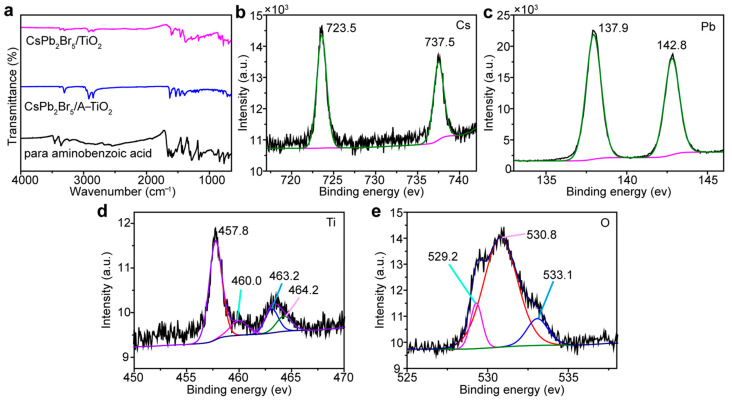
(**a**) FTIR spectra of PABA, CsPb_2_Br_5_/A-TiO_2_, and CsPb_2_Br_5_/TiO_2_; (**b**–**e**) XPS spectra of Cs3d, Pb4f, Ti2p, and O1s in CsPb_2_Br_5_/TiO_2_. Spectra are calibrated with respect to the C 1s peak (at 285.35 eV).

To better understand the formation of TiO_2_ shells of CsPb_2_Br_5_ NCs, Fourier transform infrared (FTIR) spectra of the ligand PABA, CsPb_2_Br_5_/A-TiO_2_, and CsPb_2_Br_5_/TiO_2_ NCs were obtained and shown in Figure 2a. The first FTIR spectrum corresponds to the ligand PABA, where peaks assigned to N-H vibrations can be observed at 3364 and 3459 cm^−1^ [20]. These peaks indicate the presence of the N-H functional group in PABA. Moving on to the FTIR spectrum of CsPb_2_Br_5_/A-TiO_2_, the strong C-H stretching bands at 2918 and 2846 cm^−1^ confirm the presence of PA/OAm ligands [23,24]. Additionally, the characteristic peaks assigned to N-H vibrations are still present in CsPb_2_Br_5_/A-TiO_2_, albeit with a decrease in intensity compared to PABA. This reduction in N-H peaks can be attributed to the interaction between NH_2_ and CsPb_2_Br_5_. Notably, the characteristic peak of -COOH is still not observed in this spectrum. Combining observations from the TEM analysis and FTIR spectra, it can be concluded that CsPb_2_Br_5_ NCs are indeed coated with A-TiO_2_. Upon calcination, the FTIR spectrum of CsPb_2_Br_5_/TiO_2_ shows a significant decrease in the characteristic peaks associated with different surface ligands. This decrease is attributed to decomposition of the ligands during the calcination process.

X-ray photoelectron spectroscopy (XPS) measurements were further carried out to study the constituent compositions and chemical states of CsPb_2_Br_5_/TiO_2_ NCs. The results of the XPS analysis are shown in Figure 2b–e: the signals of Pb, Cs, Ti, and O elements are clearly illustrated. To calibrate the binding energies, the reference C 1s peak at 284.6 eV was used. As shown in Figure 2b, the binding energy peaks at 737.5 and 723.5 eV can be assigned to Cs 3d_3/2_ and Cs 3d_5/2_, respectively [25,26]. Figure 2c shows the high-resolution XPS spectrum of the Pb 4f core level. The two spectral peaks with binding energies of 137.9 and 142.8 eV correspond to Pb 4f_7/2_ and Pb 4f_5/2_ levels. 

The Ti 2p peaks can be fitted into four bands at 457.8, 460.0, 463.2, and 464.2 eV, corresponding to Ti^3+^ 2p_3/2_, Ti^4+^ 2p_3/2_, Ti^3+^ 2p_1/2_, and Ti^4+^ 2p_1/2_ [27,28]. Moreover, the high-resolution spectrum of O 1s is also obtained. As shown in Figure 2e, the O 1s main peaks at 529.2 and 530.8 eV are indexed to the Ti-O bonds and Ti-OH groups, which is consistent with the binding energy of O^2−^ in the TiO_2_ lattices [29].

**Figure 3:**
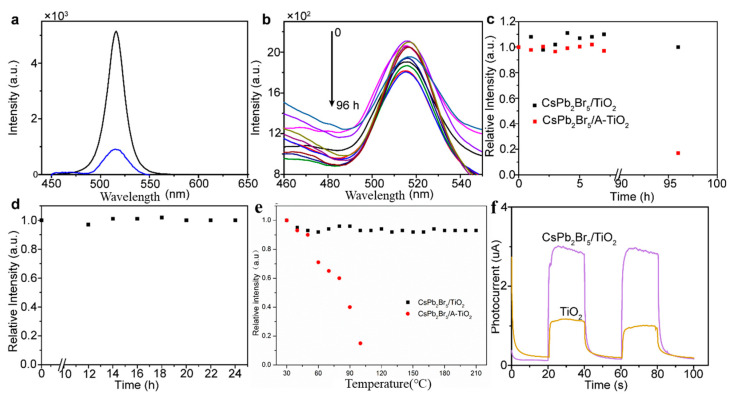
(**a**) PL spectra of perovskite before (black line) and after (blue line) the addition of TiO_2_, (**b**,**c**) PL intensity and relative PL intensity of CsPb_2_Br_5_/A-TiO_2_ and CsPb_2_Br_5_/TiO_2_ NCs powders after immersion in water, (**d**,**e**) photostability and thermal stability of CsPb_2_Br_5_/A-TiO_2_ and CsPb_2_Br_5_/TiO_2_ NCs, and (**f**) photocurrent response of TiO_2_ and CsPb_2_Br_5_/TiO_2_.

In order to investigate the influence of TiO_2_ on the PL characteristics of perovskite NCs, a comprehensive analysis of the PL spectra was conducted before and after the introduction of TiO_2_. By measuring the PL spectra, we aimed to assess any changes in the intensity, peak position, and overall emission behavior of the perovskite NCs following the addition of TiO_2_. As shown in Figure 3a, compared with perovskite NCs without TiO_2_, the PL intensity of solid CsPb_2_Br_5_/TiO_2_ NCs decreases. This decrease in PL intensity can be attributed to the transfer of electrons from the conduction band (CB) of CsPb_2_Br_5_ NCs to that of TiO_2_. This phenomenon supports the potential use of CsPb_2_Br_5_/TiO_2_ NCs in optoelectronic applications [16,30]. Hence, it is reasonable that the CsPb_2_Br_5_/TiO_2_ NCs can be used in optoelectronics. In addition, the problem of the instability of perovskite NCs often restricts their application. Thus, the environmental stability of CsPb_2_Br_5_ NCs embedded in TiO_2_, including water stability, photostability, and thermal stability, were studied. These properties are crucial for the long-term performance and reliability of optoelectronic devices utilizing CsPb_2_Br_5_/TiO_2_ NCs. The findings from these studies can provide valuable insights into the feasibility and suitability of CsPb_2_Br_5_/TiO_2_ NCs for various optoelectronic applications. 

As shown in Figure 3b,c, the PL intensity of CsPb_2_Br_5_/TiO_2_ NCs and CsPb_2_Br_5_/A-TiO_2_ NCs did not show a significant decrease within 10 h of immersion in water. It is worth noting that the water-resistance stability is much higher than that of CsPb_2_Br_5_/A-TiO_2_. When the soaking time in water was increased to 96 h, the PL intensity of CsPb_2_Br_5_/TiO_2_ NCs was essentially the same as the original PL intensity. This suggests that the water stability of CsPb_2_Br_5_/TiO_2_ NCs is maintained even after prolonged exposure to water. The PL intensity of CsPb_2_Br_5_/A-TiO_2_ NCs decreased to less than 20% that of the original PL intensity. On the other hand, the PL intensity of CsPb_2_Br_5_/A-TiO_2_ NCs decreased to less than 20% of the original PL intensity. This significant decrease in PL intensity indicates that CsPb_2_Br_5_/A-TiO_2_ NCs are not as water-stable as CsPb_2_Br_5_/TiO_2_ NCs. These results highlight the importance of the formation of TiO_2_ under calcination conditions when improving the water stability of perovskite NCs. The presence of TiO_2_ in CsPb_2_Br_5_/TiO_2_ NCs helps to enhance their resistance to water degradation. This finding suggests that the incorporation of TiO_2_ into perovskite NCs represents a promising strategy to improve their water stability. The photostability of CsPb_2_Br_5_/TiO_2_ powder was also monitored. 

As shown in Figure 3d, the photostability test result demonstrates that CsPb_2_Br_5_/TiO_2_ NCs can maintain their original PL intensity even under UV light for 24 h. Additionally, another drawback of the application of CsPb_2_Br_5_ NCs is their instability at elevated temperatures. Figure 3e indicates that when the treatment temperature was increased from 30 to 200 °C, the PL intensity of CsPb_2_Br_5_/TiO_2_ was essentially consistent with the original PL intensity; that is, the PL intensity does not decrease with increasing temperature. However, the PL intensity of CsPb_2_Br_5_/A-TiO_2_ decreased to about 10% when the temperature was increased to 100 °C. This result further indicates that the TiO_2_ shell can protect the CsPb_2_Br_5_ NCs effectively. The enhanced stability of these nanocrystals in different environmental conditions further strengthens their potential in the field. Future research efforts can focus on optimizing the stability and performance of CsPb_2_Br_5_/TiO_2_ NCs for practical applications in optoelectronics.

Based on the above discussion, the stability of CsPb_2_Br_5_ can be enhanced by coating with TiO_2_. This is due to the electrical conductivity of TiO_2_, which allows for better charge transport properties. In order to further study the photo-electrochemical characterization, a comparison was made between TiO_2_ and CsPb_2_Br_5_/TiO_2_ NCs using a 405 LED light (Zhonghe, Gauangzhou, China) in a 0.5 M K_2_SO_4_ aqueous solution. In Figure 3f, the transient photocurrent responses are shown, indicating that the cathodic photocurrent of CsPb_2_Br_5_/TiO_2_ NCs is approximately three times larger than that of pure TiO_2_. This suggests that the introduction of CsPb_2_Br_5_ enhances the photoelectronic properties of TiO_2_. Under UV light radiation, electron–hole pairs are formed in TiO_2_, allowing for photo-responsive behavior in water. However, after the introduction of CsPb_2_Br_5_, additional electrons can be formed under irradiation in CsPb_2_Br_5_ and then transported to TiO_2_. This leads to a significantly higher photocurrent in CsPb_2_Br_5_/TiO_2_ compared to pure TiO_2_. Overall, the coating of TiO_2_ on CsPb_2_Br_5_ not only enhances the stability of CsPb_2_Br_5_ but also improves its photoelectronic properties. This finding highlights the potential of CsPb_2_Br_5_/TiO_2_ NCs in various applications, such as solar cells and photocatalysis.

## 4. Conclusions

In this work, we aimed to investigate the impact of TiO_2_ on the environmental stability of CsPb_2_Br_5_. The experimental findings reveal a significant enhancement in the stability of CsPb_2_Br_5_ against various environmental factors, including water, heating, and photo radiation. This improvement in stability can be attributed to the incorporation of TiO_2_. Furthermore, encapsulating CsPb_2_Br_5_ NCs into anatase TiO_2_ (which has excellent electrical conductivity) enables the photoinduced charge in perovskite to transfer efficiently. Compared to pure TiO_2_, CsPb_2_Br_5_/TiO_2_ shows higher photoelectric activity in water testing. These results have important implications for the development of optoelectronic devices and visible light photocatalysts. The incorporation of TiO_2_ not only enhances the stability of CsPb_2_Br_5_ but also improves its photoelectric performance. This work provides valuable insights and guidelines for the preparation of advanced optoelectronic materials.

Future research into these new nanocrystals (NCs) should be directed towards exploring their vast potential in optoelectronic applications. Specifically, researchers should concentrate on harnessing the unique properties of these NCs to develop advanced technologies such as lasers, light-emitting diodes (LEDs), photovoltaics, optical sensing systems, and photon detection devices.

## Data Availability

Data are contained within the article.

## Supplementary Materials

The following supporting information can be downloaded at https://www.mdpi.com/article/10.3390/mi14122186/s1. Figure S1: EDS pattern of the CsPb_2_Br_5_/TiO_2_. Figure S2: XRD patterns of a) CsPb_2_Br_5_ and b) TiO_2._ Figure S3: SAD images of the (a) CsPb_2_Br_5_/A-TiO_2_ and (b) CsPb_2_Br_5_/ TiO_2._
