# Supplementary material for: Encapsulation of CsPb2Br5 in TiO2 Microcrystals to Enhance Environmental Stability
Source: Micromachines (Basel). 2023 Nov 30;14(12):2186. doi: 10.3390/mi14122186 (PMC10745879; doi:10.3390/mi14122186)
Supplement: Supplementary file 1 [file micromachines-14-02186-s001.zip › micromachines-2720365-supplementary.pdf]

Supplementary Materials

Encapsulation of $CsPb_2Br_5$ in TiO_2 Microcrystals to Enhance Environmental Stability

Yuezhu Wang, Xiaotong Xu *, Wenchao Yang, Yawen Wei and Junsheng Wang

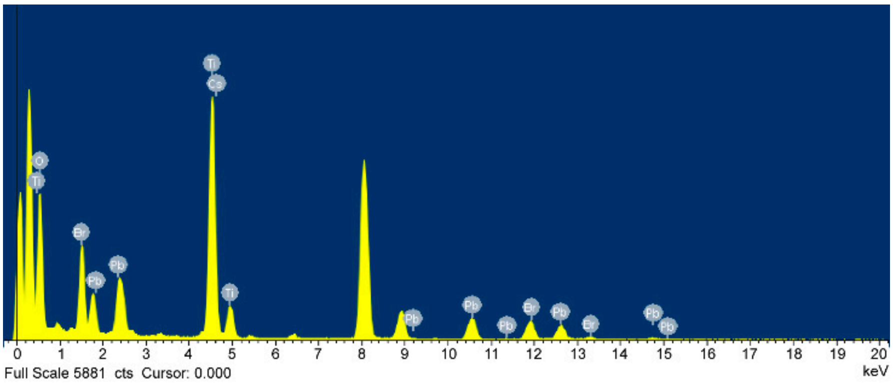

Figure S1. EDS pattern of the $CsPb_2Br_5/TiO_2$.

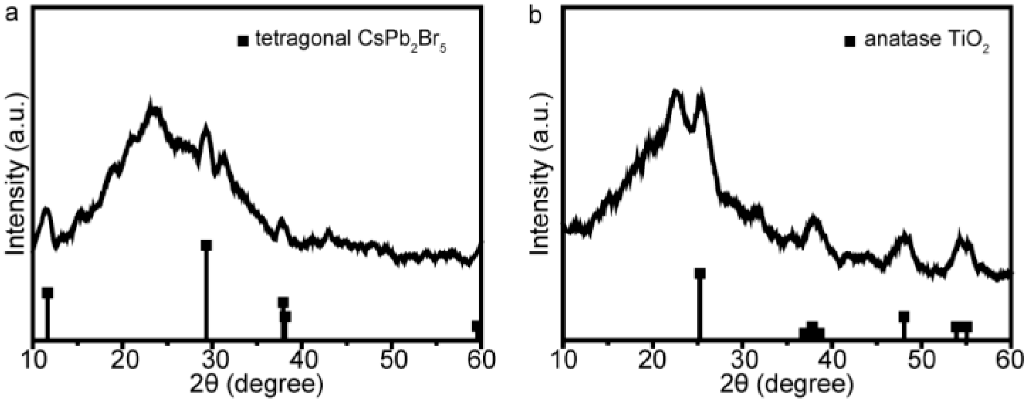

Figure S2. XRD patterns of (**a**) $CsPb_2Br_5$ and (**b**) TiO_2.

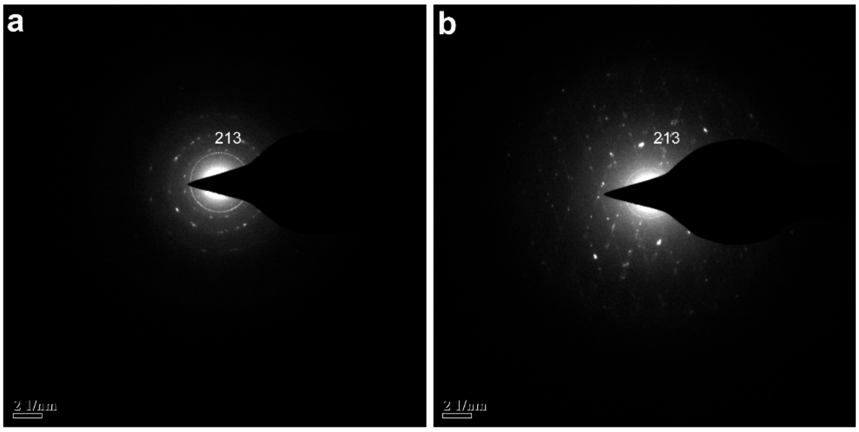

Figure S3. SAD images of the (**a**) $CsPb_2Br_5$/A-TiO_2 and (**b**) $CsPb_2Br_5$/ TiO_2
